# WWP-1 Is a Novel Modulator of the DAF-2 Insulin-Like Signaling Network Involved in Pore-Forming Toxin Cellular Defenses in *Caenorhabditis elegans*

**DOI:** 10.1371/journal.pone.0009494

**Published:** 2010-03-02

**Authors:** Chang-Shi Chen, Audrey Bellier, Cheng-Yuan Kao, Ya-Luen Yang, Huan-Da Chen, Ferdinand C. O. Los, Raffi V. Aroian

**Affiliations:** 1 Department of Biochemistry and Molecular Biology, National Cheng Kung University, Tainan, Taiwan; 2 Institute of Basic Medical Sciences, National Cheng Kung University, Tainan, Taiwan; 3 Section of Cell and Developmental Biology, University of California San Diego, La Jolla, California, United States of America; Duke University Medical Center, United States of America

## Abstract

Pore-forming toxins (PFTs) are the single largest class of bacterial virulence factors. The DAF-2 insulin/insulin-like growth factor-1 signaling pathway, which regulates lifespan and stress resistance in *Caenorhabditis elegans*, is known to mutate to resistance to pathogenic bacteria. However, its role in responses against bacterial toxins and PFTs is as yet unexplored. Here we reveal that reduction of the DAF-2 insulin-like pathway confers the resistance of *Caenorhabditis elegans* to cytolitic crystal (Cry) PFTs produced by *Bacillus thuringiensis*. In contrast to the canonical DAF-2 insulin-like signaling pathway previously defined for aging and pathogenesis, the PFT response pathway diverges at 3-phosphoinositide-dependent kinase 1 (PDK-1) and appears to feed into a novel insulin-like pathway signal arm defined by the WW domain Protein 1 (WWP-1). In addition, we also find that WWP-1 not only plays an important role in the intrinsic cellular defense (INCED) against PFTs but also is involved in innate immunity against pathogenic bacteria *Pseudomonas aeruginosa* and in lifespan regulation. Taken together, our data suggest that WWP-1 and DAF-16 function in parallel within the fundamental DAF-2 insulin/IGF-1 signaling network to regulate fundamental cellular responses in *C. elegans.*

## Introduction

Pore-forming toxins (PFTs) are bacterial toxins that damage the plasma membrane of host cells and play important roles in the pathogenesis of many important human pathogens including *Staphylococcus aureus, Streptococcus pyogenes, Clostridium perfringens,* and *Enterococcus faecalis* [1], [2], [3]. Crystal (Cry) toxins produced by the Gram-positive spore-forming soil bacterium *Bacillus thuringiensis* (Bt) are a large family of PFTs [4]. Over 100 phylogenetically related three-domain Cry toxins are known [4]. Although known primarily for their ability to kill insects [5], we have reported that some Cry toxins, including Cry5B and Cry21A, can intoxicate a wide range of plant-parasitic, animal-parasitic, and free-living nematodes including the standard laboratory nematode species, *Caenorhabditis elegans* [6], [7], [8].

This *C. elegans*–Cry toxin interaction system opened up the first, and to date the only, whole-animal genetic model for studying PFTs *in vivo* and led to the discovery of signal transduction pathways that protect cells against PFTs, including the p38 mitogen-activated protein kinase (MAPK) pathway and the unfolded protein response (UPR) pathway, which are also protective against and/or activated by PFTs in mammalian cells [9], [10], [11]. More recently, we have shown that the hypoxia response pathway also protects *C. elegans* against PFTs [12]. Although significant and extensive responses to PFTs at the molecular level (*e.g.*, modulation of signal transduction cascades) have been recorded, the roles of these responses in coping with PFTs as yet poorly understood [13]. Thus, functional analyses of innate immune responses to the single largest class of bacterial protein virulence factors remains a largely understudied area.

The DAF-2 insulin/insulin-like growth factor 1 (IGF-1) receptor pathway is part of a neuroendocrine system that regulates longevity, metabolism, and development in *C. elegans* and is homologous to the mammalian insulin and IGF-1 signaling pathway [14], [15]. The *C. elegans daf-2* gene encodes the worm homolog of the insulin/IGF-1 receptor. *C. elegans* strains that carry reduction-of-function or loss-of-function mutations in *daf-2* or the downstream phosphoinositol 3-kinase (PI3K) *age-1* are long-lived and are also resistant to a variety of stresses and bacterial pathogens [16], [17], [18].

Here we demonstrate that reduction of the DAF-2 insulin/IGF-1 signaling pathway confers resistance to Bt Cry PFTs in *C. elegans*. Unexpectedly, this resistance does not solely rely on the canonical DAF-2 insulin/IGF-1 signaling pathway through AKT/PKB and DAF-16 but that rather, at least in part, deviates form the main pathway at PDK-1 and that includes WWP-1. We demonstrate that WWP-1 may be a novel signaling arm that diverges from PDK-1 and functions in parallel to DAF-16 in the DAF-2 insulin/IGF-1 signaling network. Furthermore WWP-1 is functionally important for the intrinsic cellular defenses (INCED) against pathogenic attacks since loss of this pathway leads to animals hypersensitive to PFTs and pathogenic bacteria *Pseudomonas aeruginosa* in *C. elegans*.

## Results

### Reduction of the DAF-2 Insulin-Like Receptor Signal Confers Resistance to Bt Cry PFTs in *C. elegans*

**Figure 1:**
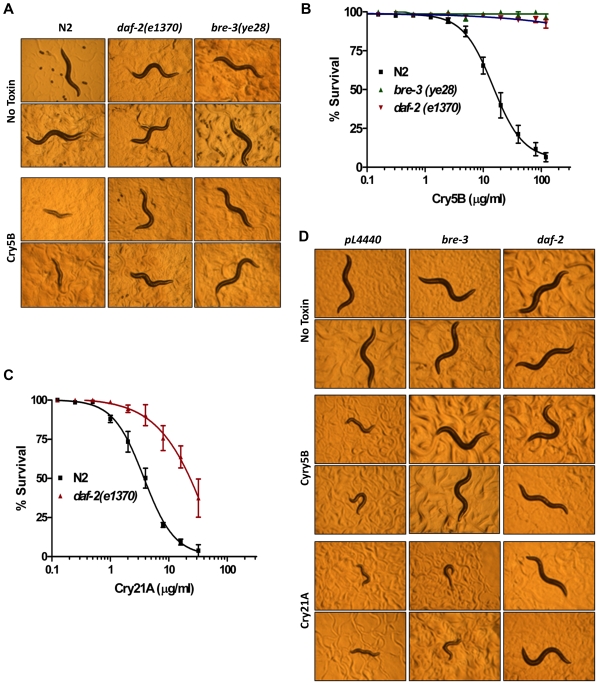
Reduction of the DAF-2 insulin-like receptor signal confers resistance to Cry toxins. (A) Comparison of the reduction-of-function *daf-2(e1370)* mutant animals to wild-type N2 animals and the known Cry5B resistant *bre-3(ye28)* animals on Cry5B-expressing *E. coli* plates indicates *daf-2(e1370)* animals are resistant to Cry5B. The experiment was performed three times. Two representative worms are shown for each strain 48 hours after feeding either on *E. coli* with (Cry5B) or without Cry5B (No Toxin). (B) A dose-dependent mortality assay was performed using purified Cry5B toxin to quantitatively compare sensitivities of wild-type N2 to the *bre-3(ye28)* and *daf-2(e1370)* mutants. Lethality was determined after 6 days. This semi-log graph represents three independent experiments, and each data point is the mean and standard deviations of the experiments. Note, that although *daf-2* mutant animals are highly resistant based on their ability to stay alive, they clearly are not as resistant as *bre-3* animals since the latter are alive and robustly active and healthy whereas the former are alive but sickly at the end of this 6 day assay. (C) The dose-dependent mortality assay for Cry21A spore-crystal lysates. The fact that spores are present in these assays is due to the fact we currently do not have purified Cry21A. The presence of spores increases the toxicity of the Cry protein. Hence, the curves in (B) and (C) cannot be directly compared. (D) RNA interference *E. coli*-expressed Cry5B toxin plate assay. RNAi sensitive *rrf-3(pk1426)* worms after feeding on *pL4440* (empty vector control), *bre-3,* or *daf-2 genes* dsRNA expressing *E. coli* were transferred to the *E. coli*-expressing either Cry5B (Cry5B), Cry21A (Cry21A) or without toxin (No Toxin) bacterial lawns for 48 hours. Knock down of the *daf-2* gene leads to resistant to both Cry5B and Cry21A toxins. The experiment was performed three times and two representative worms are shown for each RNAi knock down strain.

To address whether the DAF-2 insulin/IGF-1 signaling pathway plays a role in *C. elegans* against the PFTs, *C. elegans daf-2* reduction-of-function mutant *daf-2(e1370)* were qualitatively compared to wild-type N2 animals in their susceptibilities to the nematicidal PFT, Cry5B (Figure 1A). Fourth larval (L4) stage worms were fed for 48 hours either on control plates with *E. coli* strain JM103 that did not express Cry5B or plates prepared with *E. coli* JM103 expressing Cry5B. Specifically, the relative health of each worm was evaluated qualitatively by comparing body size, darkness of the intestine as an indicator of feeding, and activity, including pharyngeal pumping and whether the worm demonstrated spontaneous movement. In the absence of Cry5B, the wild-type and mutant worms are healthy adults with similar appearance. In the presence of the Cry5B, wild-type N2 worms were severely intoxicated compared to those found on control no-toxin plates, as evidenced by their smaller sizes and paler appearances (Figure 1A). However, under the same conditions, the *daf-2(e1370)* mutant animals were qualitatively healthier and appeared resistant to Cry5B.

The sensitivity to Cry5B PFT of animals mutant for the DAF-2 insulin/IGF-1 receptor was also quantitatively assessed using a dose-dependent mortality assay (Figure 1B) [19]. Form these data, LC_50_ (lethal concentration at which 50% of the animals die) values were obtained (Table 1). These quantitative results confirm that *daf-2(e1370)* mutant animals are at least an order of magnitude more resistant to Cry5B than wild-type N2 animals (P<0.01). In order to test whether *daf-2(e1370)* mutant animals were also resistant to another Cry PFT, we also quantitative analyzed the LC_50_ values of *daf-2(e1370)* animals to the nematicidal Cry PFT, Cry21A [7], [12]. These data show that *daf-2(e1370)* animals are 7X more resistant to Cry21A than wild type N2 animals (Figure 1C and Table 1; P<0.01).

**Table 1:** Data analysis of the quantitative Crystal toxins lethal concentration assays.

Toxin and Strain	LC_50_ (µg/ml)†	Standard Deviation	p value relative to N2	Relative sensitivity (LC_50_ mutant/LC_50_ N2)
Cry5B LC_50_ assay (Figure 1B)				
N2 wild-type	11.24	1.86		
*bre-3(ye28)*	>120§	§	<0.01	>10
*daf-2(e1370)*	>120	§	<0.01	>10
Cry21A LC_50_ assay (Figure 1C)				
N2 wild-type	3.73	1.03		
*daf-2(e1370)*	26.10	8.03	p<0.01	7.00
Cry5B LC_50_ assay (Figure 2D)				
N2 wild-type	10.42	1.09		
*daf-2(e1370)*	>120	§	p<0.01	>10
*pdk-1(sa680)*	>120	§	p<0.01	>10
*pdk-1(sa709)*	>120	§	p<0.01	>10
*pdk-1(mg142)*	7.19	0.76	p<0.05	0.69
Cry5B LC_50_ assay (Figure 2E)				
N2 wild-type	20.55	6.64		
akt-1(ok525)	10.21	1.40	p<0.05	0.50
akt-1(sa573)	5.77	0.82	p<0.01	0.28
akt-2(ok393)	18.30	1.96	0.589	0.89
sgk-1(ok538)	13.95	1.49	0.133	0.68
Cry5B LC_50_ assay (Figure 3C)				
N2 wild-type	7.44	1.45		
*wwp-1(ok1102)*	1.33	0.15	p<0.01	0.18
*pcm-1(qa201)*	6.46	1.92	0.428	0.87
Cry5B LC_50_ assay (Figure 3D)				
N2 wild-type	8.33	1.09		
*wwp-1(gk372)*	2.52	1.03	p<0.01	0.30
*wwp-1(gk397)*	1.90	0.77	p<0.01	0.23
*wwp-1(ok1102)*	1.30	0.26	p<0.01	0.16

†The range of the LC_50_ values for Cry5B in N2 animals varies from 8.33 to 20.55 µg/ml, because the absolute quality and measured quantity of toxins varies from different fermentation batches. We performed each experiment using the same batch of the purified toxins.

§The absolute LC_50_ values of these mutant worms can not be calculated by Probit, because the limitation of the solubility of Cry5B and therefore we cannot achieve a 100% killing concentration for these mutants. We used 120 µg/ml, the maximum dose of Cry5B, for the statistical calculations of these resistant mutants.

To independently confirm the importance of DAF-2 in PFT responses, DAF-2 function was reduced using RNA interference (RNAi) in the RNAi-sensitive strain *rrf-3(pk1426)* (Figure 1D). The resistant to Cry5B and Cry21A resulting from knockdown of *daf-2* was also seen, confirming the Cry PFT resistant phenotype is caused by reduction of DAF-2 function. Interestingly, knock down of *bre-3* gene induced resistance to Cry5B but not to Cry21A at the level detectable by this qualitative assay, although more quantitative data indicate that mutants lacking *bre-5* that functions in the same pathway as *bre-3* show partial resistance to Cry21A (data not shown). This result implies that Cry21A at least partly might require a different receptor than Cry5B for intoxication of *C. elegans*.

### Resistance of *daf-2* Mutant to Cry Toxins Is, in Part, through a *daf-16*-Independent Manner

**Figure 2:**
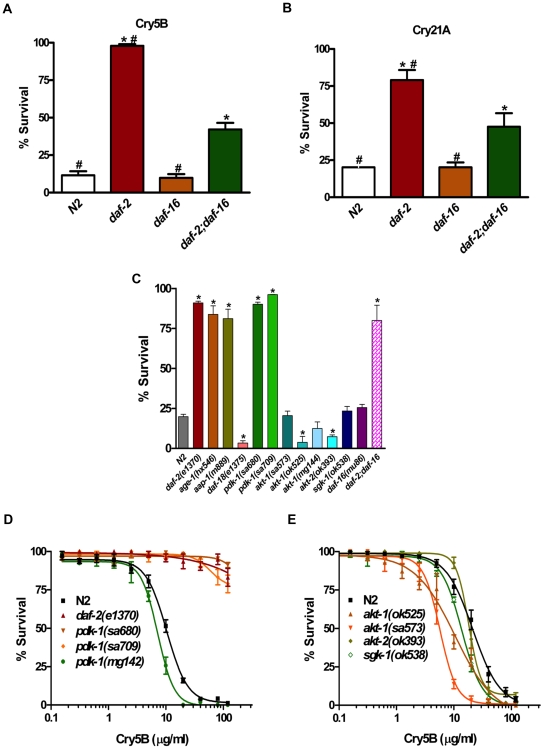
The resistance to Cry toxin is only partly dependent upon DAF-16 FOXO and forks at PDK-1 in the DAF-2 insulin-like network. (A, B) Comparisons of the *daf-2(e1370), daf-16(mu86), and daf-2(e1370);daf-16(mu86)* mutant animals to wild-type N2 animals in 40 µg/ml purified Cry5B or 8 µg/ml Cry21A indicate *daf-2(e1370)* mutant and *daf-2(e1370);daf-16(mu86)* double mutant are all statistically resistant to Cry5B and Cry21A compared with N2. * indicates P<0.001 (P<0.05 in B) relative to wild-type N2. ^#^ indicates P<0.001 (P<0.05 in B) relative to *daf-2(e1370);daf-16(mu86)* mutant. (C) Comparisons of the mutants in the canonical DAF-2 insulin-like signaling pathway, including *daf-2(e1370), age-1(hx546), aap-1(m889), daf-18(e1375), pdk-1(sa680), pdk-1(sa709), akt-1(sa573), akt-1(ok525), akt-1(mg144), akt-2(ok393), sgk-1(ok538), daf-16(mu86), daf-2(e1370);daf-16(mu86)*, to wild-type N2 animals in *E. coli*-expressed Cry5B liquid toxicity assay. * indicates P<0.01 relative to wild-type N2. (D, E) Dose-dependent mortality were performed using purified Cry5B toxin to quantitatively compare sensitivities of wild-type N2 to the *daf-2(e1370), pdk-1(sa680), pdk-1(sa709), pdk-1(mg142), akt-1(ok525), akt-1(sa573), akt-2(ok393), and sgk-1(ok538)* mutants. This semi-log graph represents three independent experiments, and each data point shows the mean and standard deviations from three experiments.

In fertile animals, the extended lifespan and enhanced stress and pathogen resistance phenotypes associated with reduction-of-function mutations in *daf-2* are totally suppressed by loss-of-function mutations in *daf-16*, which encodes a Forkhead transcription factor [17], [20], [21], [22], [23]. To test whether DAF-16 is also functionally downstream of DAF-2 with regards to Cry PFT resistance, animals containing the partial lost-of-function *daf-2(e1370)* mutant, the *daf-16* null mutant, *mu86* [24], and the *daf-2(e1370);daf-16(mu86)* double mutant were exposed to Cry5B and Cry21A and scored for viability at the single dose of 40 µg/ml purified Cry5B [8]. Under these conditions, *daf-2(e1370)* animals are significantly more resistant (8.5X more alive animals) to Cry5B than wild-type N2 animals (P<0.01) whereas loss-of-function *daf-16(mu86)* mutant animals are as sensitive as N2 (Figure 2A, Table 2; P = 0.683). *daf-2(e1370);daf-16(mu86)* double mutant animals had an intermediate phenotype that was statistically more sensitive than *daf-2(e1370)* animals (P<0.001) but statistically more resistant (respectively 4.3X and 3.7X more alive animals) than *daf-16(mu86)* or wild-type animals in response to Cry5B (P<0.001 and P<0.001). Similar results were also seen in the animals treated with 8 µg/ml of the Cry21A. *daf-2(e1370);daf-16(mu86)* double mutant animals also had an intermediate phenotype that was statistically more sensitive than *daf-2(e1370)* animals (P<0.05) but statistically more resistant (respectively 2.3X and 2.4X more alive animals) than *daf-16(mu86)* or wild-type animals in response to Cry21A (P<0.05 and P<0.05) (Figure 2B, Table 2). These results indicate that DAF-16 is required for some, but not all, of the PFT resistance conferred by reduction of DAF-2 function.

**Table 2:** Data analysis of the toxins toxicity assays.

Cry5B 40 µg/ml (Figure 2A)	Survival (%)	Standard Deviation	p value relative to N2	p values relative to *daf-2;daf-16*
N2 wild-type	11.52	9.47		p<0.001
*daf-2(e1370)*	97.91	3.45	p<0.001	p<0.001
daf-16(mu86)	9.81	7.72	p = 0.663	p<0.001
*daf-2(e1370);daf-16(mu86)*	42.16	10.69	p<0.001	

Cry21A 8 µg/ml (Figure 2B)	Survival (%)	Standard Deviation	p value relative to N2	p values relative to *daf-2;daf-16*
N2 wild-type	20.21	2.67		P<0.05
*daf-2(e1370)*	79.07	11.65	p<0.01	P<0.05
daf-16(mu86)	20.23	5.59	p = 0.996	P<0.05
*daf-2(e1370);daf-16(mu86)*	47.51	15.93	p<0.05	

*E. coli-*expressed Cry5B liquid toxicity assay (Figure 2C)	Survival (%)	Standard Deviation	p value relative to N2	Relative sensitivity (mutant/wild type)
N2 wild-type	19.82	3.44		
*daf-2(e1370)*	90.91	1.90	p<0.01	4.59
*age-1(hx546)*	83.75	9.32	p<0.01	4.23
*aap-1(m889)*	81.08	8.39	p<0.01	4.09
*daf-18(e1375)*	3.34	2.04	p<0.01	0.17
*pdk-1(sa680)*	90.15	1.80	p<0.01	4.55
*pdk-1(sa709)*	96.11	0.13	p<0.01	4.85
*akt-1(sa573)*	20.53	3.94	p = 0.822	1.04
*akt-1(ok525)*	3.76	5.32	p<0.01	0.19
*akt-1(mg144)*	12.50	5.73	p = 0.08	0.63
*akt-2(ok393)*	7.41	1.48	p<0.01	0.59
*sgk-1(ok538)*	23.44	4.00	p = 0.279	1.18
*daf-16(mu86)*	25.61	2.77	p = 0.092	1.29
*daf-2(e1370);daf-16(mu86)*	79.91	13.59	p<0.01	4.03

### The Resistance of Cry Toxins Signal in DAF-2 Signaling Pathway in Part Deviates from PDK-1

In the canonical DAF-2 insulin/IGF-1 signaling pathway for the regulation of lifespan, energy metabolism, and dauer development in *C. elegans*, DAF-2 regulates DAF-16 through the activation of PI3K, encoded by *age-1* and *aap-1* for the catalytic subunit and the regulatory subunit respectively. PI3K potentiates the activity of PDK-1, which in turn activates the three downstream serine/threonine kinases: the Akt/PKB homologs AKT-1 and AKT-2, and the serum- and glucocorticoid-inducible kinase homolog, SGK-1. These three serine/threonine kinases than inhibit the nuclear translocation and transcriptional activity of DAF-16 by phosphorylating DAF-16 at different serine/threonine residues [25]. In order to identify where in the canonical DAF-2 insulin-like signaling pathway a DAF-16-independent arm might branch off for responding to Cry PFTs, we obtained available mutants in this pathway from the *Caenorhabditis* Genetic Center (CGC) and exposed them to *E. coli* expressing Cry5B in liquid medium. In this Cry5B toxicity assay, the *daf-2(e1370), age-1(hx546), aap-1(m889), pdk-1(sa680), pdk-1(sa709),* and *daf-2(e1370);daf-16(mu86)* animals are all statistically resistant to Cry5B compared to the wild-type N2 animals (Figure 2C, Table 2). Consistent with these results, *daf-18(e1375)* mutant animals (DAF-18 encodes the mammalian PTEN lipid phosphatase homolog that antagonizes PI3K), are hypersensitive to Cry5B compare to wild-type animals. Thus, animals from the insulin-receptor down to PDK-1 behave as expected from what is known about the pathway. However, animals with mutations in the three serine/threonine kinases immediately downstream of PDK-1, including *akt-1(sa573), akt-1(ok525), akt-1(mg144), akt-2(ok393),* and *sgk-1(ok538),* were not resistant to Cry5B (Figure 2C, Table 2), and in fact in two cases (*akt-1(ok525)* and *akt-2(ok393)*) were hypersensitive to the PFT.

To independently test these results, the sensitivity to Cry5B of animals with mutations in the *pdk-1, akt-1, akt-2* and *sgk-1* genes were quantitatively assessed using the Cry5B dose-dependent mortality assay (Figure 2D and 2E). Our quantitative results comparing LC_50_ values confirmed that animals with reduction-of-function mutations in *pdk-1*, including *pdk-1(sa680)* and *pdk-1(sa709),* were statistically more resistant to Cry5B than wild type N2 animals. A gain-of-function mutation in *pdk-1(mg142)* was, as predicted, sensitive to Cry5B. As above, we found animals with the loss-of-function mutations in *akt-1, akt-2* and *sgk-1*, including *akt-1(sa573), akt-1(ok525), akt-2(ok393),* and *sgk-1(ok538)* were either as sensitive or hypersensitive to Cry5B compared to N2 animals (Figure 2D and Table 1). Since there is some redundancy between *akt-1*, *akt-2*, and *sgk-1* mutant animals, it is possible that the triple mutant (which is lethal and therefore not possible to test; [26]) might be partly resistant. However, it should be noted that *sgk-1* mutant animals on their own have a long-lived phenotype [27]. The fact that *sgk-1* animals were not resistant to Cry5B and that no level of resistance was seen with *akt-1* or *akt-2* suggested that there is a divergence of the insulin pathway with regards to Cry5B response upstream of these genes and branching off at PDK-1.

### WWP-1 Is a Novel PDK-1 Interacting Protein Involved in Cry5B Defense

**Figure 3:**
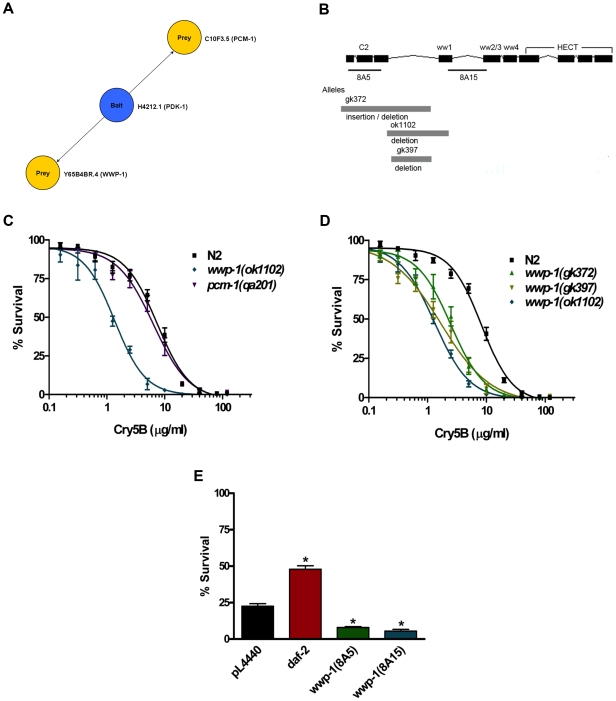
WWP-1 is a novel PDK-1 interacting protein involved in Cry5B defense. (A) Two novel PDK-1 interacting protein identified by the Worm Interactome Database. Two bait-prey interactions, including H42K12.1(*pdk-1*)-Y65B4BR.4(*wwp-1*) and H42K12.1(*pdk-1*)-C10F3.5(*pcm-1*), were identified by using H42K12.1(*pdk-1*) as the bait in the high-throughput yeast two hybrid screens [28]. (B) Predicted genomic structure of *wwp1*. Boxes and lines denote exons and introns respectively. The predicted functional domains of WWP1, including a C2 domain, four WW domains and a HECT domain, are indicated. The regions corresponding to the interfering dsRNA clones, 8A5 and 8A15 are underlined. The mutation regions of the three *wwp-1* mutant alleles used in this study are indicated as gray boxes. (C) A Cry5B dose-dependent mortality assay was performed to quantitatively compare sensitivities of wild-type N2 to the *wwp-1(ok1102)* and *pcm-1(qa201)* mutants. Only *wwp-1(ok1102)* mutant animals showed statistically (p<0.01) hypersensitivity to Cry5B compared to N2. (D) A Cry5B dose-dependent mortality assay was performed to quantitatively compare sensitivities of wild-type N2 to three *wwp-1* mutants, including *wwp-1(gk372), wwp-1(gk397), wwp-1(ok102)*. All *wwp-1* mutants showed statistically hypersensitive to Cry5B. (E) RNAi sensitive *rrf-3(pk1426);glp-4(bn2)* mutant worms after feeding on *pL4440* (empty vector control), *daf-2,* or two *wwp-1*(8A5 and 8A15) dsRNA expressing *E. coli* were exposed to purified Cry5B at 25°C. Lethality was determined after 6 days. Animals knocked down for the *daf-2* gene were statistically resistant to Cry5B whereas animals knocked down for the *wwp-1* gene by two independent *wwp-1* RNAi clones were statistically hypersensitive to Cry5B.

In order to identify the novel signaling arm that branches off from PDK-1, we searched the *C. elegans* Interactome database (http://vidal.dfci.harvard.edu/interactomedb/i-View/interactomeCurrent.pl) for novel PDK-1 interacting proteins [28]. Two novel PDK-1 interacting proteins, WWP-1(WW domain Protein) and PCM-1(Protein CarboxyMethyltransferase), were identified from high-throughput yeast two-hybrid screens using H42K12.1(PDK-1) as the bait (Figure 3A). The *wwp-1* gene encodes a putative E3 ubiquitin ligase orthologous to budding yeast Rsp5, Drosophila Su(dx), and human WWP1 and WWP2 [29] (Figure 3B), and the *pcm-1* gene encodes an L-isoaspartate O-methyltransferase orthologous to human PCMT1 [30]. We requested all available mutants of these two genes from CGC and exposed them to Cry5B. The sensitivity to Cry5B of animals with mutations in the *wwp-1* and *pcm-1* genes were quantitatively assessed using the Cry5B dose-dependent lethality assay and LC_50_ values were obtained (Figure 3C and Table 1). The results showed that *wwp-1(ok1102)* loss-of-function mutant animals are significantly hypersensitive to Cry5B PFT compared to wild type N2 animals (>6 fold; P<0.01), but the sensitivity to Cry5B PFT of *pcm-1(qa201)* mutant animals are statistically indistinguishable from N2 animals. Two additional *wwp-1* mutant alleles, *wwp-1(gk372)* and *wwp-1(gk397)* (Figure 3B), were also tested. All three *wwp-1* mutant alleles are significantly hypersensitive to Cry5B PFT (Figure 3D, Table 1).

To independently test that our results stem from loss of function phenotypes, RNAi sensitive *rrf-3(pk1426);glp-4(bn2)* mutant worms were used to knock down the *wwp-1* genes by two independent *E. coli* RNAi feeding clones (8A5 and 8A15) from the Ahringer RNAi library [31]. *rrf-3(pk1426);glp-4(bn2)* animals were used because the *rrf-3(pk1426)* mutant is hypersensitive to RNAi and the *glp-4(bn2)* mutant does not produce progeny that would otherwise complicate the assay (via internal hatching of larvae that sometimes occurs when adult *C. elegans* are intoxicated with Cry proteins). We have demonstrated that both mutants have roughly normal response to Cry5B [10]. DNA sequencing results demonstrated that these two *wwp-1* RNAi clones target different sequence regions of the *wwp-1* gene (Figure 3B). The resistant and hypersensitive phenotypes to Cry5B resulting from knockdown of *daf-2* and *wwp-1* respectively were seen, confirming the phenotypes are caused by lost or reduction of function of these genes (Figure 3E, Table 3). Overall, these data suggest that WWP-1, a novel PDK-1 putative interacting protein, is functionally important for defense against PFT attack since loss of this pathway leads to animals hypersensitive to Cry5B PFT.

**Table 3:** Data analysis of the RNA interference assays.

RNAi in *rrf-3(pk1426);glp-4(bn2)* (Figure 3E)	Survival (%)	Standard Deviation	p value relative to RNAi empty vector control (pL4440)	
pL4440 (vector control)	22.55	3.00		
*daf-2* RNAi (pAD48)	47.91	4.11	p<0.01	
*wwp-1* RNAi (8A5)	7.97	0.98	p<0.01	
*wwp-1*RNAi (8A15)	5.43	2.15	p<0.01	

*wwp-1* RNAi (Figure 6)	Survival (%)	Standard Deviation	p value relative to RNAi empty vector control (pL4440)	Relative sensitivity (*wwp-1* RNAi/vector control)	p values relative to *daf-2;daf-16/wwp-1* RNAi
N2/pL4440	41.87	12.25			0.12
N2/*wwp-1*	4.84	5.32	p<0.01	0.12	0.06
*daf-2*/pL4440	99.22	1.35			
*daf-2*/*wwp-1*	75.87	5.52	p<0.01	0.76	
*daf-2;daf-16*/pL4440	69.21	1.62			
*daf-2;daf-16/wwp-1*	23.15	11.23	p<0.01	0.33	
*wwp-1*/pL4440	8.06	2.17			
*wwp-1/wwp-1*	6.25	3.27	p = 0.471	0.78	

### WWP-1 Is also Involved in Innate Immunity and Aging Regulation

**Figure 4:**
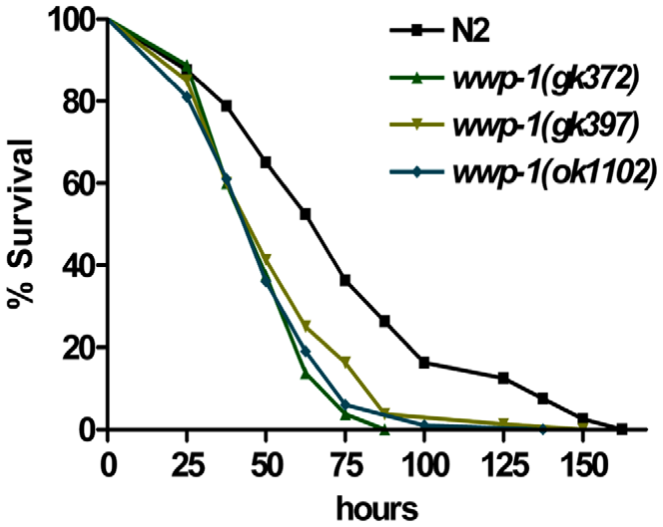
WWP-1 is involved in the innate immunity against *P. aeruginosa* PA14. A lifespan assay was used to compare the *wwp-1* mutants to slow killing by *P. aeruginosa* PA14. This graph represents combined data from three experiments. The lifespan of *wwp-1* mutants, including *wwp-1(gk372)* and *wwp-1(ok1102)*, feeding on *P. aeruginosa* PA14 bacteria are statistically shorter than wild-type N2 animals.

The DAF-2 signaling network in *C. elegans* is well documented for essential in innate immunity against various pathogens, including the human Gram-negative bacterial pathogens *Pseudomonas aeruginosa* [17], [18]. If WWP-1 is in the DAF-2 pathway and is antagonistic to DAF-2 function as it is relative to the Cry protein response (*i.e.*, DAF-2 animals are resistant whereas WWP-1 mutant animals are hypersensitive), we predict WWP-1 would also play a role in response to *P. aeruginosa* and be antagonistic to DAF-2. We exposed *wwp-1* mutants to the pathogenic bacteria *P. aeruginosa* strain PA14 (Figure 4 and Table 4). As we predicted, animals lacking WWP-1 are significantly hypersensitive to the killing by *P. aeruginosa* PA14 (P<0.01; the opposite phenotype of DAF-2 mutant animals). These data also demonstrate that WWP-1 is involved in promoting innate immunity against pathogenic bacteria.

**Table 4:** Data analysis of the lifespan and general stresses assays.

Life span assay (Figure 5A) and strain	Median survival (days)	p value relative to N2	
N2 wild-type	17.00		
*wwp-1(gk372)*	12.00	p<0.01	
*wwp-1(ok1102)*	10.50	p<0.01	

CuSO_4_ (Figure 5B) and strain	LC_50_ (mM)	Standard Deviation	p value relative to N2
N2 wild-type	2.59	0.57	
*wwp-1(gk372)*	3.55	0.93	0.094
*wwp-1(gk397)*	3.45	0.93	0.146
*wwp-1(ok1102)*	1.93	0.39	0.306

H_2_O_2_ (Figure 5C) and strain	LC_50_ (mM)	Standard Deviation	p value relative to N2
N2 wild-type	1.26	0.25	
*wwp-1(gk372)*	1.57	0.42	0.144
*wwp-1(gk397)*	1.62	0.39	0.076
*wwp-1(ok1102)*	1.38	0.28	0.788

PA14 killing assay (Figure 4) and strain	Median survival (hours)	p value relative to N2	
N2 wild-type	75		
*wwp-1(gk372)*	50	p<0.01	
*wwp-1(gk397)*	50	p<0.01	
*wwp-1(ok1102)*	50	p<0.01	

**Figure 5:**
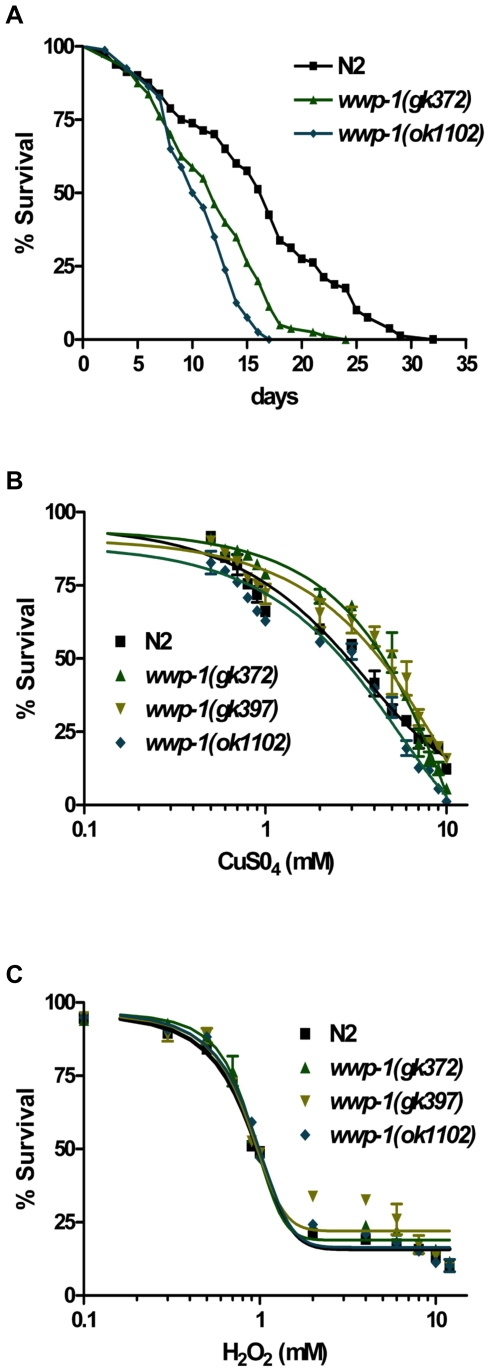
WWP-1 is a positive regulator of lifespan in *C. elegans*. (A) A lifespan assay was used to compare the normal lifespan of *wwp-1* mutant worms and the wild-type N2 worms. This graph represents combined data from three independent experiments. The lifespan of *wwp-1* mutants, including *wwp-1(gk372)* and *wwp-1(ok1102)*, are statistically shorter than wild-type N2 animals. (B) A dose-dependent mortality assay comparing sensitivity to CuSO_4_ revealed the *wwp-1* mutants, *wwp-1(gk372), wwp-1(gk397),* and *wwp-1(ok1102)* are not hypersensitive compared to wild-type N2. Lethality was determined after 6 days of CuSO_4_ exposure, the same time frame as the Cry5B lethality assay. Data, plotted semi-log, are the mean and standard deviation of three independent experiments. (C) A dose- dependent mortality assay comparing sensitivity to H_2_O_2_ revealed *wwp-1* mutants are not hypersensitive to this toxic insult compared to wild-type N2. Lethality was determined after 4 hours of H_2_O_2_ exposure. Data, plotted semi-log, are the mean and standard deviation of three independent experiments.

The DAF-2 insulin/IGF-1 signaling network as aforementioned also plays important roles in regulation of lifespan in *C. elegans*. It also has been suggested that signal transductions in the DAF-2 insulin/IGF-1 signaling pathway for longevity and innate immunity can be interrelated [16], [18]. If WWP-1 is in the DAF-2 pathway, it might also have a role in *C. elegans* lifespan, again antagonistic to DAF-2. As predicted, we found that *wwp-1(gk372)* and *wwp-1(ok1102)* mutant animals had statistically significant shorter lifespan compared with wild-type N2 animals (Figure 5A, Table 4). These data indicate that *wwp-1* is also a positive regulator of lifespan in *C. elegans.*

To confirm that the hypersensitivity of WWP-1 mutant animals to aging, *P. aeruginosa*, and Cry proteins is not due to general ill health of these animals, we tested whether *wwp-1* mutant animals are hypersensitive to two other toxic chemical compounds, the heavy metal CuSO_4_ (a toxic insult that kills with kinetics similar to Cry5B) [11] and the oxidative stress agent H_2_O_2_ (a toxic insult that kills much more rapidly). All *wwp-1* mutants, including *wwp-1(gk372), wwp-1(gk397),* and *wwp-1(ok1102)*, have the similar sensitivity as wild type to killing by either CuSO_4_ or H_2_O_2_ (Figure 5B and 5C; Table 4). These data argue against the supposition that these mutants are hypersensitive to the PFT and *P. aeruginosa* tested above merely because they are generally unhealthy. Taken together, the above results suggest that WWP-1 is not only a positive regulator in the longevity regulation but also specifies in the intrinsic cellular defense (INCED) against PFTs and the innate immune response against pathogenic bacteria in *C. elegans*.

### WWP-1 Works Downstream of DAF-2 and in Parallel to DAF-16 in the DAF-2 Insulin/IGF-1 Signaling Network

**Figure 6:**
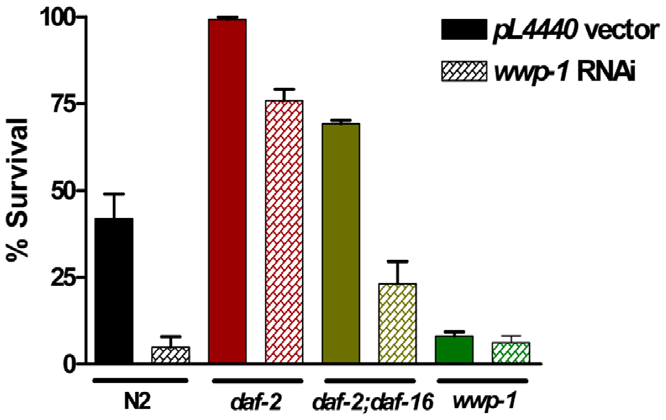
WWP-1 is a downstream signal of DAF-2 and in parallel to DAF-16 in response to Cry5B. *daf-2(e1370), daf-16(mu86), daf-2(e1370);daf-16(mu86) and wwp-1(ok1102)* mutant worms were fed dsRNA to wwp-1 (clone 8A15) to knock down *wwp-1* gene in all these strains (plaid boxes). The worms fed on *E. coli* HT115 transformed with the RNAi empty vector (*pL4440* vector) were used as controls (solid boxes). After developing to L4 stage, all RNAi knock down worms are exposed to purified Cry5B at 25°C. Lethality was determined after 6 days. Knockdown of *wwp-1* gene in animals, including N2, *daf-2(e1370), daf-16(mu86),* and *daf-2(e1370);daf-16(mu86)*, increased their sensitivity to Cry5B statistically significant. However, the sensitivity of *wwp-1(ok1102)* animals to Cry5B cannot be further enhanced by *wwp-1* RNAi.

The two-hybrid interactome data and the Cry5B/aging/*P. aeruginosa* results are consistent with WWP-1 acting in the DAF-2 pathway antagonistic to and downstream of DAF-2. If true, then we predict that knock-down of WWP-1 should suppress a DAF-2 mutant phenotype. We therefore knocked down *wwp-1* in *daf-2(e1370)* and *daf-2(e1370);daf-16(mu86)* mutant animals and exposed these *wwp-1* knockdown animals (and control “no knock down” animals in which the animals were fed empty RNAi vector) to purified Cry5B (Figure 6, Table 3). Our results showed that *wwp-1* RNAi can partly suppress the *daf-2(e1370)* resistant phenotype and that the combination of *daf-16* knock out (via *mu86* mutation) and *wwp-1* knock down (via RNAi) can completely suppress *daf-2(e1370)* resistance back to a response similar to wild-type (p = 0.12 *daf-2;daf-16*/wwp-1 RNAi vs. N2/L4440 vector control; note, although the response of *daf-2;daf-16*/wwp-1 RNAi animals looks less hypersensitive than that of N2/wwp-1 RNAi animals, the two are actually statistically similar; P = 0.06). These data are consistent with both *daf-16* and *wwp-1* acting in parallel daf-2-dependent pathways to mediate resistance to Cry5B PFT. Furthermore, that *wwp-1* RNAi can not further sensitize *the wwp-1(ok1102) worms* in this experiment also confirmed that this mutant allele is a total loss-of-function mutant.

## Discussion

**Figure 7:**
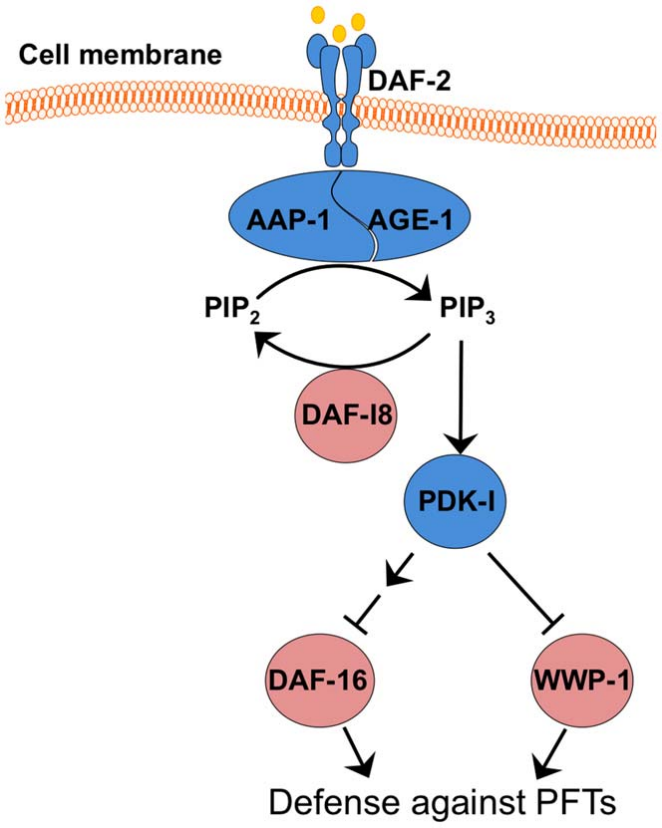
Schematic illustrating relationship between WWP-1 and DAF-2 insulin-like signal network. In the canonical DAF-2 insulin/IGF-1 signaling pathway in *C. elegans*, DAF-2 regulates DAF-16 through the activation of PI3K, composed of AGE-1 and AAP-1 for the catalytic subunit and the regulatory subunit respectively. PI3K activates the activity of PDK-1, which eventually inhibits the nuclear translocation and transcriptional activity of DAF-16. Here we demonstrate that the attenuated DAF-2 signal can also regulate an additional DAF-16-independent signal arm that diverges form PDK-1 to WWP-1 to defense against Cry5B PFT.

Here we demonstrate for the first time that the DAF-2 insulin/IGF-1 pathway is involved in intrinsic cellular defenses (INCED) against PFTs. Furthermore, our data suggest that the *daf-2* PFT defense pathway bifurcates at PDK-1 into DAF-16-dependent and DAFT-16-independent branches. This is the first report of a bifurcation of the daf-2 insulin/IGF-1 pathway at this junction. We furthermore find a protein, WWP-1, that appears to be involved in the DAF-16 –independent branch of the INCED/innate immune response. Loss of WWP-1, a protein in the ubiquitin E3 ligase family, leads to hypersensitivity to the PFT Cry5B and the pathogen *P. aeruginosa* and that, based on double and triple knock out/knock down analyses with *daf-2* and *daf-16* mutants, appears to work in parallel with DAF-16. A model summarizing our findings is shown in Figure 7.

The assignment of WWP-1 as downstream of the DAF-2 insulin/IGF-1 pathway is based on two-hybrid interactome data with PDK-1, on phenotypic analyses of responses to PFT, *P. aeruginosa*, and aging, and on our genetic pathway data. A similar conclusion is evidenced from a previous genome-wide RNAi screen in which it was shown that knockdown of *wwp-1* gene decreased median lifespan by 24% in *daf-2(e1370)* mutants, 5% in *daf-2(e1370);daf-16(mgDf47*) mutants, and 9% in wild-type N2 animals [32]. The knockdown of *wwp-1* gene more significantly shortened the lifespan of *daf-2* animals but still decreased the lifespan of *daf-2;daf-16* mutant and wild-type N2 animals. This suggested that *wwp-1* functions in parallel to *daf-16* and converging within the DAF-2 insulin/IGF-1 signaling network to regulate longevity, which is reminiscent of our data. In addition, recently ubiquitin ligase activity has been shown for WWP-1, as well as demonstration that WWP-1 is involved in a *daf-16*-independent life span extension in response to diet restriction [33]. Taken together, our and others' results imply that WWP-1 functions in parallel to DAF-16 and converging within the fundamental DAF-2 insulin/IGF-1 signaling network to regulate the INCED against PFTs attack, innate immune responses against *P. aeruginosa*, as well as longevity in *C. elegans*.

Our results also show that regulation of PFT defense and lifespan can clearly be decoupled. First, we note that lifespan extension of *daf-2* mutants is completely dependent upon *daf-16* [34] but response to PFT is not. Second, we note that where *sgk-1* mutants are long-lived [27], mutation in this gene alone is not resistant to PFTs. The hypersensitivity of akt-1 and possibly akt-2 mutant animals is intriguing and suggests that they might also have other roles outside of the DAF-2 insulin/IGF-1 pathway in PFT defenses. Interestingly along these lines, we note that in mammalian cells a role of AKT in response to PFTs has already been noted [35].

That DAF-2 signal can be decoupled from the conical pathway is reminiscent of several recent reports. Firstly, it has been demonstrated that EAK-3 (Enhancer of AKT-1 null) functions in parallel to AKT-1 to inhibit the expression of Forkhead transcription factor DAF-16 target genes involved in *C. elegans* dauer development. That Eak-3 mutants have normal lifespan indicates EAK-3 decouples insulin-like regulation of development and longevity [36], [37]. Secondly, it has been reported that long-lived mutants of genes downstream of *daf-2*, such as *pdk-1* and *sgk-1*, show wild-type resistance to the human opportunistic pathogen, *Pseudomonas aeruginosa* strain PA14. However, mutants of *akt-1* and *akt-2* show enhanced resistance to *P. aeruginosa* PA14 [26]. Thirdly, it has also bee reported that some *akt-1* and *pdk-1* alleles can uncouple the dauer arrest, adult longevity and stress resistance phenotypes of *age-1(mg109)* mutants [38]. They also demonstrated reproductive development in *age-1(mg109); mg227* animals required only *akt-1*, and *pdk-1* activity was dispensable in this background. These findings suggested larval and adult phenotypes of DAF-2 signaling are fully separable in these mutants. Finally, the other possible PDK-1 interacting protein identified in the interactome database, PCM-1, has also been demonstrated to participate in the repair of age-damaged proteins and overexpression of PCM-1 increases adult life span [39], [40]. However in our results, PCM-1 is not involved in Cry5B INCED. All of these data suggest that dauer formation, lifespan regulation, stress response, and pathogen resistance signals can be intertwined and in some cases decoupled within the DAF-2 insulin/iGF-1 signaling network in *C. elegans*.

In summary, we have identified specifically WWP-1 and the DAF-2 insulin/IGF-1 signaling network as components of INCED against PFTs. The biological functions of the DAF-2/DAF-16 signaling pathway in longevity and pathogens resistance have been extensively studied for years [15], [18], [41]. Our current hypothesis is that the DAF-2 signaling network has split off a separate arm at PDK-1/WWP-1 not only involved in longevity regulation and the innate immunity against *P. aeruginosa* PA14 but also the INCED against PFTs as well as perhaps some yet unknown responses.

## Materials and Methods

### *C. elegans* and Bacterial Strains

Some *Caenorhabditis elegans* strains used in this work were provided by the *Caenorhabditis* Genetics Center (CGC), which is funded by the NIH National Center for Research Resources (NCRR). *C. elegans* strains were maintained on NG plates using *Escherichia coli* strain OP50 as the food source [42]. Strains used in this study were wild-type Bristol strain N2, *daf-2(e1370), age-1(hx546), aap-1(m889), daf-18(e1375), pdk-1(sa680), pdk-1(sa709), pdk-1(mg142), akt-1(ok525), akt-1(sa573), akt-1(mg144), akt-2(ok393), sgk-1(ok538), daf-16(mu86), daf-2(e1370);daf-16(mu86), bre-3(ye28), rrf-3(pk1426), rrf-3(pk1426); glp-4(bn2), pcm-1(qa201), wwp-1(ok1102), wwp-1(gk372),* and *wwp-1(gk397)* were each backcrossed at least 4 times. Bacteria expressing dsRNA directed against *bre-3* and *wwp-1* were part of a *C. elegans* RNAi library in *E. coli* strain HT115 (Geneservice, Cambridge U.K.). All RNAi clones have been confirmed by plasmid DNA sequencing. *Escherichia coli* HT115 transformed with the *p*AD48 construct, which expresses dsRNA targeting the *daf-2* gene, was kindly provided by A. Dillin (Salk Institute, San Diego) [43]. All bacterial strains were cultured under standard conditions.

### Cry Toxins Toxicity Assays and Microscopy

All assays were performed at 25°C unless indicated elsewhere. Qualitative toxicity assays based on visual comparison of worm intoxication were performed on plates with *E. coli*-expressed Cry5B as described [19]. L4 stage worms were fed for 48 hours either on control plates with *E. coli* that did not express Cry5B (pQE9 vector control) or on plates prepared with *E. coli* expressing Cry5B (pQE9-Cry5B) [7]. The relative health of each worm was determined qualitatively by comparing body size, darkness, and activity. Images were acquired using an Olympus SZ60 compound microscope using the 3X magnification linked to a Canon PowerShot A620 digital camera. Quantitative mortality assays were performed as described [19]. Concentrations of each toxin were set-up in triplicate for each assay with approximately 20∼30 worms per well, and each assay was performed independently at least three times. The purified Cry5B and the crystal-spore toxin lysates of Cry21A were prepared as described [8], [44]. Approximately 1500 worms were scored for each strain in the calculation of the LC_50_ values for each toxin. *E. coli*-expressed Cry5B liquid toxicity assay: N2 wild-type and various *daf-2* pathway mutant worms were exposed to *E. coli*-expressed Cry5B in S media in 48-wll plates to quantitative scored the survival. Similar conditions were used as the quantitative mortality assays described above, except that *E. coli* JM103 Cry5B expressing bacteria at 0.6 OD600 in stead of purified Cry5B and *E. coli* OP50 were used in this assay. The survival rate of each well was scored after incubating at 25°C for 6 days.

### RNA Interference (RNAi)

For figures 1D, RNAi assays were carried out on *E. coli*-expressed Cry5B Plates. *E. coli* strain HT115 transformed with RNAi plasmids were spread on NG-IC plates [NG plates with 25 µg/ml carbenicillin and 0.1 mM isopropyl-β-D-1-thiogalactopyranoside (IPTG)] and incubated at 25°C overnight to induce the dsRNA expression. *E. coli* HT115 with *p*L4440, an empty vector, was used as negative control of RNAi. Synchronized *rrf-3(pk1426)* L1 larvae were obtained using standard protocols [19] then cultured on *pL4440*, *bre-3,* or *daf-2 genes* dsRNA expressing RNAi-plates at 20°C until L4 stage. These L4 stage worms were transferred to either control plates with *E. coli* HT115 that did not express Cry toxins (empty vector) or plates prepared with *E. coli* HT115 expressing either Cry5B or Cry21A (using our standard expression vector; [7]) together with *E.coli* HT115 either carrying RNAi plasmids or the *p*L4440 plasmid and then incubated at 25° for 48 h. The relative health of each worm was determined qualitatively by its appearance as described above.

For figures 3E, synchronized L1 *rrf-3(pk1426);glp-4(bn2)* animals (mutant is hypersensitive to RNAi and does not produce progeny that would otherwise complicate the assay; both mutants have roughly normal response to Cry5B; [10]) were cultured on the NG-IC *daf-2* or *wwp-1* RNAi-plates at 20°C until L4 stage. L4 stage *rrf-3(pk1426);glp-4(bn2)* RNAi knock down animals were washed out by S medium and transferred to the wells of 48-well plate with S medium containing *E. coli* HT115 RNAi bacteria at 0.6 OD600 and 20 µg/ml of purified Cry5B. After incubating at 25°C for 6 days, the survival rate of each well was scored.

For figure 6, the *daf-2(e1370), daf-16(mu86), daf-2(e1370);daf-16(mu86) and wwp-1(ok1102)* mutant worms were used. Synchronized L1 animals were cultured on the NG-IC *wwp-1* RNAi or pL4440 *E. coli* HT115 plates at 20°C until L4 stage. L4 stage RNAi knock down animals were washed out by S medium and transferred to the wells of 48-well plate with S medium containing with either *wwp-1* RNAi or pL4440 *E. coli* HT115 bacteria at 0.6 OD600 and 20 µg/ml of purified Cry5B. After incubating at 25°C for 6 days, the survival rate of each well was scored.

### Lifespan Assay

Lifespan analysis was conducted according to standard protocols [34], [45]. All life span experiments were performed in the absence of 5-fluoro-2′-deoxyuridine. Briefly, to obtain a synchronously growing population, eggs were prepared by treating a population of *C. elegans* with hypochlorite/NaOH solution and transferring the resulting eggs to NG agar plates covered with *E. coli* strain OP50. When these had reached the young adults, ∼150 nematodes were transferred to fresh plates, which also represents the first day of life span analysis. Nematodes were transferred to fresh plates daily during the progeny production period and after that were transferred every second to third day but monitored daily for dead animals. Nematodes that did not respond to gentle prodding and displayed no pharyngeal pumping were scored as dead. Animals that crawled off the plate or died due to internal hatching or protrusion of the gonads through the vulva were censored. Censoring describes an event where partial information on the life span of an individual animal is lost as a consequence of premature death. Thus, censored animals were included in statistical analysis only until the day of the censoring event. Survival analysis was performed using GraphPad Prism 5.0 (GraphPad Software, Inc. La Jolla, CA). The Mantel-Cox logrank test was used to assess statistical significance of difference in survival. Only p-values<0.01 were considered significant to minimize type I errors.

### Pseudomonas aeruginosa PA14 Killing Assay

The *P. aeruginosa* killing assay was performed on slow-killing plates as described [46], with the following modifications: PA14 was cultured overnight in tryptic soy broth instead of King's broth and then spread on slow-killing plates complemented with 75 uM 5-fluoro-2′-deoxyuridine. The experiment was performed three times with approximately 100–150 worms total per strain at 25°C. The Mantel-Cox logrank test was used to assess statistical significance of difference in survival. Only p-values<0.01 were considered significant to minimize type I errors.

### General Stressors Analysis

#### CuSO_4_ assay

Assays were carried out as described [11]. In brief, a serial doses of CuSO_4_, *E. coli* OP50 at an optical density of 0.2–0.25 OD600, and ∼30 L4 larvae were used per well in 48-well plates. Lethality was determined after 6 days of CuSO_4_ exposure at 25°C.

#### H_2_O_2_ assay

Assays were carried out as described [11]; lethality was determined after 4 hours of H_2_O_2_ exposure at 25°C.

### Data Analysis

All experiments were performed a minimum of three times. LC_50_ values were determined by PROBIT analysis [47]. The lethal concentration assays are represented graphically using nonlinear regression performed with the software GraphPad Prism 5.0. Statistical analysis between two values was compared with a paired t-test. Statistical analysis among three or more values was compared with one-way ANOVA with Dunnett adjustment. All data analysis was performed using SPSS, ver 13.0 (SPSS, Chicago, IL). Statistical significance was set at p<0.05.
